# An overview of insulin therapy for the non‐specialist

**DOI:** 10.1111/dom.16280

**Published:** 2025-03-04

**Authors:** Philip D. Home

**Affiliations:** ^1^ Translational and Clinical Research Institute Newcastle University Newcastle upon Tyne UK

**Keywords:** biosimilars, diabetes care, insulin, insulin therapy, primary care, secondary care, type 1 diabetes, type 2 diabetes

## Abstract

Nearly all health professionals, whatever their practice or speciality, now have contact with a significant number of insulin‐using people with diabetes. People with type 1 diabetes are nearly universally managed on more complex insulin regimens, increasingly with complex support technology, and with some understanding of the concepts underlying these needed by anyone with responsibility for other aspects of their health care. People with type 2 diabetes usually come to insulin therapy in time, now with a prevalence for insulin administration of ≈50%, thanks to improved management of associated health risks giving longer life expectancy. But while some understandings have simplified the usual approach (basal insulin, self‐adjustment dose algorithms), the advent of other medical products offering cardio‐renal protection, the use of insulin in combination with these, and the marketing of novel insulin analogues and biosimilars, have all added complexity to clinical decision making. However for the insulin user in the mainstream of care (ambulatory diabetes services in primary and secondary care) the broad principles of choice and management of insulin therapy are fairly easily applied. In the current article, the complexity is dissected and addressed, some of the stigma around insulin therapy is neutralised, and the fundamental approach in regular care drawn into focus.

**Plain Language Summary:**

Insulin therapy is used in their diabetes lifetime by nearly all people with type 2 diabetes (T2DM), as well as in all people with type 1 diabetes (T1DM). In T2DM this is in the context of the usual progression of islet B‐cell secretory failure, but also very often in the context of other conditions, from cancer or steroid therapy to acute arterial events or major surgery. Its prescription in these circumstances means that, in pharmaco‐epidemiological studies, insulin use is invariably associated with poor health outcomes, but in a series of major RCTs of 5–15 years duration no excess of vascular or oncological adverse health outcomes was found. Physiologically insulin secretion occurs in two scenarios, namely basal insulin at night and between meals (≈50%), and in short (≈4 h) bursts with meals (≈50%). The former suppresses liver glucose production, which in its absence causes plasma glucose to rise threefold to around 12 mmol/l (but much higher if metabolic stress or with sugar‐containing drinks). Meal‐time insulin secretion in addition promotes glucose storage in skeletal muscle. People with T1DM, having no endogenous insulin secretion, thus require a multiple injection regimen (basal + meal‐time), or pumped insulin, often now moderated by continuous glucose monitoring (CGM). Basal and meal‐time preparations of pharmaceutical insulin analogues are also used for T2DM, with GLP‐1RA and metformin usually continued. The starting regimen is normally basal only, usually with insulin glargine (100 U/ml), originator or biosimilar, while insulin degludec or glargine 300 U/ml can have advantage as basal insulins where true 24‐h cover is found to be needed. Weekly insulins (developmental or marketed) may have a role in injection acceptability in T2DM, at a cost of some increase in hypoglycaemia. In ambulatory care a meal‐time insulin analogue, originator or biosimilar, is added when required, often after some years on basal insulin. Meal insulins are also used in insulin pumps. Hypoglycaemia is a significant issue in T1DM, limiting insulin dosing, but can be helped by CGM, with or without pumps, and careful dose adjustment. It is a much lesser issue in T2DM, until meal‐time insulins are introduced, or in people of thinner phenotype, whence again expertise in dose adjustment may be needed. Body weight gain with insulin is usually modest, particularly if basal insulin is begun appropriately before glycosuria has influenced calorie balance. In mainstream ambulatory care the broad principles of insulin therapy are fairly easily applied, the main resources being team familiarity with a basal insulin (glargine/biosimilar), a finger‐prick glucose‐monitoring system, basic patient education on use of these, and time to supervise dose titration. Beginning with a fixed dose (e.g. 10 U/day) and increasing by 2 U twice a week is simple, but may take months of persistent input to reach target fasting plasma glucose levels. In conclusion, insulin is a usual therapy in both T1DM and T2DM, and in the latter initially at least is fairly easily applied, in combination with other glucose‐lowering agents. However it can be more challenging in the context of the technology used in T1DM, once meal‐time insulin is added to basal in T2DM, and when dose requirements are complex and unstable in conjunction with other medical conditions.

## 1 INTRODUCTION

### 1.1 A brief epidemiology

The general epidemiology of diabetes is well‐rehearsed.^1^ But in the context of insulin therapy it is worth noting that <5% of people with diabetes have type 1 diabetes (T1DM), despite their generally earlier onset of the condition (≈50% <18 years) and their generally long life expectancy.^2^, ^3^ The global epidemic of type 2 diabetes (T2DM) continues,^1^ and, with dominant incidence still in middle age or later, lifetime risk while varying geographically is now typically 20%–50%. Since people with T2DM generally come to insulin 5–15 years after diagnosis, and with life expectancy of over 20 years or often longer, over 50% will be on insulin therapy at any one time. This is particularly true for those with multiple medical conditions, and therefore notably the older individual, as insulin is often indicated where other medical management is more complicated, from cancer to renal failure to heart disease. As a result an increasing proportion of the total managed population, whether in primary care or secondary care, or being seen in other specialties as in‐patients or out‐patients, will be taking insulin therapy.

The associations between circulating insulin concentrations, insulin therapy, and adverse health outcomes have undeservedly served insulin badly. Early observations, in non‐diabetic people, that higher plasma insulin concentrations were associated with cardiovascular disease, are now understood to be confounding with the adverse features of the insulin‐resistant metabolic syndrome such as dyslipidaemia, dysglycaemia, vascular inflammation, hypercoagulability, and high blood pressure.^4^ Insulin therapy has been the therapy of choice in people with complex medical conditions beyond their diabetes, from renal failure to cirrhosis to cancer therapy and many others, such that its use is always associated with poorer life expectancy in comparative pharmaco‐epidemiological observational studies. However, larger, long, clinical trials in which insulin therapy is compared to other diabetes therapies simply do not support any such adverse effects.^5^, ^6^, ^7^, ^8^, ^9^ Meanwhile mechanistic studies find insulin therapy reduces glucotoxicity and lipotoxicity, ameliorates insulin resistance, benefits carotid intima‐media thickness, and has various desirable endothelial and anti‐inflammatory properties.^10^, ^11^, ^12^, ^13^ There is little or no residual concern.

There have been suggestions that the growth factor (anabolic) properties of insulin could be tumour‐supportive. A particular issue arose over insulin glargine, as the native product in the injection solution has a higher growth factor to glucose‐lowering activity than human insulin. However, the circulating product after subcutaneous absorption does not, indeed the opposite.^14^, ^15^ Again the longer outcome studies give no hint of a problem.^6^, ^9^ Further insulin has been injected in very high concentrations subcutaneously for over 100 years in hundreds of millions of people without a single report of an injection‐site neoplasm.

### 1.2 Insulin and type of diabetes

It is not absolutely necessary to know the type or pathogenetic origin of an individual's diabetes to manage their insulin therapy. This is because management, including choice of preparations used and insulin dose requirement, is in practice determined by glucose control as measured by self‐monitoring. Indeed, particularly in the early years after diagnosis of type 2 diabetes, it can be unclear what type of diabetes is being managed (Table 1).^16^ While specific tests, notably plasma C‐peptide, can help, these have large ranges of uncertainty, while the auto‐immune markers of type 1 diabetes can be positive in type 2 diabetes,^17^ and middle‐aged people with abdominal obesity can develop T1DM. Much type 2 diabetes in thinner people beyond retirement age is unclassifiable. What is often labelled as type 2 diabetes, including for clinical trial purposes, contains a rump of genetic syndromes previously known as maturity‐onset diabetes of the young, while latent autoimmune diabetes of adults, generally sits with type 2 diabetes clinically but is aetiologically closer to type 1 diabetes.^17^ In general, many types of maturity‐onset diabetes of the young respond well to sulfonylureas,^18^ so in the past, before sulfonylureas became unfashionable, the need for insulin was simply determined by glucose control, even in the absence of specific diagnosis. An issue can arise, however, with genetic syndromes if diabetes is diagnosed in childhood, perhaps at a time of metabolic stress causing glucose levels to rise markedly, whence the paediatric diabetes team may conservatively assume T1DM, and start insulin with a plan for reviewing that need. This also occurs with neonatal diabetes, and, very rarely nowadays, where glucosuria is misconfirmed as diabetes.

**TABLE 1:** Clinical types of diabetes and how they map to insulin therapy.

Diabetes type	Comment	Insulin requirement
Type 1 diabetes—autoimmune	Progresses rapidly to essentially complete insulin deficiency; slower progression if adult onset	Insulin‐dependent, basal and meal‐time insulin, acute ketoacidosis without insulin
Neonatal diabetes	Usually congenital single enzyme deficiency	Sulfonylurea responsive after genetic confirmation
Maturity onset diabetes of the young	Multiple genetic syndromes; testing available	Majority respond to sulfonylurea therapy
Secondary pancreatic diabetes	Variable degrees of pancreatic loss	Varies from insulin‐dependent to desirable; can be insulin sensitive (low doses) and hypoglycaemia prone
Type 2 diabetes	Progressive insulin deficiency usually with marked insulin resistance	Progresses over years to basal insulin need, then with addition of meal‐time insulin
	LADA ‘sub‐type’, or thin older people	More rapid need for insulin therapy and in lower doses
Gestational diabetes; pregnancy in type 2 diabetes	Tighter glucose control targets	Early use of insulin if indicated by glucose control
Endocrine diabetes	Cushing's syndrome, acromegaly, steroid therapy	Often a requirement for insulin therapy

*Note*: This table omits rarer diabetes syndromes (e.g., lipodystrophic and mitochondrial) and complex interactions with other medical conditions and therapies (e.g., myocardial infarction, antipsychotics, and checkpoint inhibitors).

Abbreviation: LADA, latent auto‐immune diabetes of adults.

To repeat, in many individuals the certain classification of the disease does not per se affect insulin management. However, clear understanding can be important to people living with diabetes. Notably of course if this is T1DM, or diabetes secondary to pancreatic disease, then uninterrupted insulin dosing will be required for life (together with reassurance as to how this can deliver a long and healthy life). In these people the limitations of insulin therapy also mean that they are hypoglycaemia prone—again it is useful to know that is usual. But similarly when starting insulin in people with T2DM in ambulatory care (as opposed to stress environments such as in‐patients), and including those with possible latent auto‐immune diabetes of adults, the point should be honestly made that this is for life, that it will benefit future health, and that doses and types of insulin will change over the years.^19^ It is also important to communicate early on, particularly for the majority with abdominal obesity started on a basal insulin, that hypoglycaemia is possible but unlikely.

## 2 INSULIN TYPES AND FORMULATIONS

### 2.1 Physiological insulin secretion

Fundamentally there are only two types of insulin on the market, meal‐time insulin and basal insulin (Table 2). Concordantly, in non‐diabetic people, there are only two scenarios of insulin secretion: basal at night and in longer meal intervals, and meal‐time.^20^, ^21^ In modern practice then, the physiological foundations of insulin therapy are logical. But this pattern of insulin secretion and pharmaceutical insulin absorption was only established from around 1980—prior to this insulin preparations were used on the basis of clinical experience.^22^

**TABLE 2:** A simple schema of clinically available insulins.

Insulin preparation/formulation	Comment
Extended‐acting insulins (basal)	
Basal insulin secretion (endogenous)	50% of physiological in non‐diabetic people; high in insulin‐resistant states, including T2DM
NPH insulin	Not a 24‐h insulin, and with a peak action at 4–6 h
Insulin glargine 100 U/mL	Gives coverage for up to 24 h, but most exposure in the first 12 h. Has become the foundation block of injected insulin regimens. Biosimilars available
Insulin detemir	Can give coverage for 24 h in higher doses, but most action in first 12 h
Insulin glargine 300 U/mL Insulin degludec	True 24‐h insulins. Injection any time of day. Minor reduction in hypoglycaemia
Insulin icodec Insulin efsitora alfa	Developmental weekly injectables. Increase in hypoglycaemia, especially in T1DM. Icodec licensed in some markets
Meal‐time insulins	
Meal‐time insulin secretion (endogenous)	Very fast rise to peak physiologically, and fall to basal levels in 4 h (Figure 1). Blunted slow climb in secretion in T2DM
Human unmodified	Slow to peak and 8‐h duration of action
Insulins lispro, aspart, and glulisine	Faster to peak, and shorter duration of action. Meal‐time insulins of choice. Biosimilars available Also basal insulin supply from pumps
Faster insulin aspart, faster insulin lispro	Demonstrable faster effect, but of marginal advantage clinically
Premix insulins	Fixed combinations of a meal‐time insulin (human, lispro, aspart) and an extended‐acting insulin (NPH, NPH type, degludec) Limit flexibility in optimizing dose titration

*Note*: Animal extracted insulins are available in some markets, as meal insulins and NPH formulations. Purity of these extracted preparations can vary.

Abbreviations: T1DM, type 1 diabetes; T2DM, type 2 diabetes.

**FIGURE 1:**
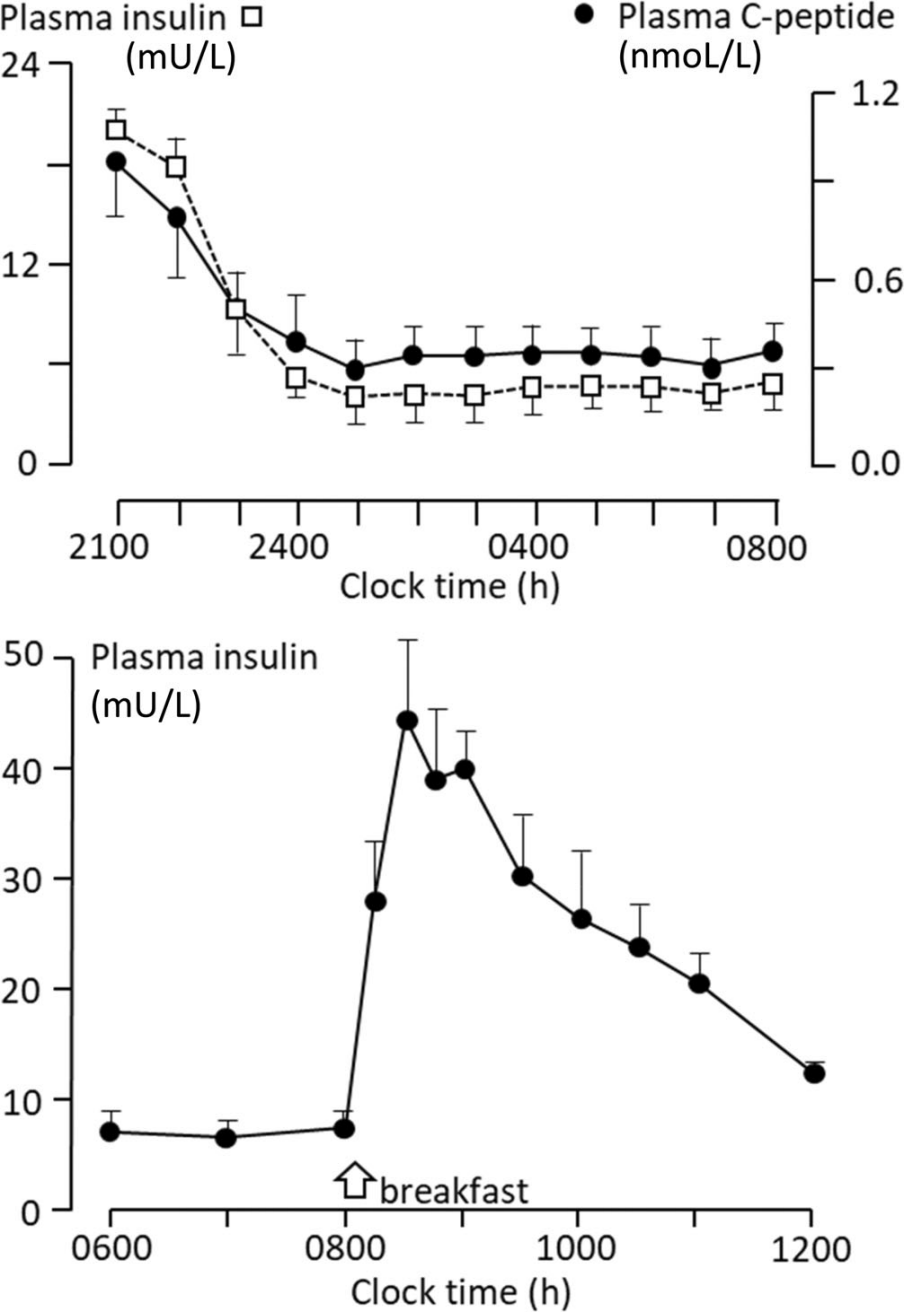
Physiological insulin secretion rate (as plasma insulin and plasma C‐peptide) in non‐diabetic fit people overnight (upper panel), and after a full breakfast (lower panel).^20^ Because insulin is rapidly cleared from the circulation through its receptor, circulating insulin concentrations show the pattern of insulin secretion. Note the constant basal secretion and clearance rates overnight (upper panel), but a very rapid rise in response to incretins after breakfast (lower panel), which is largely ameliorated by 4 h. Insulin therapy in people with diabetes should seek to emulate these patterns, if optimal glucose control is to be achieved.

The physiological pattern is illustrated in Figure 1—a perfectly flat basal secretion overnight, with a very rapid rise at meal times to a peak from 30 min, with a steady fall again to basal levels at around 4 h.^20^, ^21^ However, in people with above‐normal circulating glucose levels insulin requirement can drift up in the later part of the night, and in all of us gastric emptying (and thus insulin need) will vary with the quantity and nature of food consumed.

Control of basal insulin secretion is through the pancreatic islet B‐cell, a quantitative sensor of circulating glucose concentration. Control of meal‐time insulin secretion is principally via incretins and reflexes initially,^23^ with modulation by circulating glucose and other nutrients with time. High circulating levels of glucose and fatty acids (glucotoxicity, lipotoxicity) can affect both the response of the islet B‐cell and the sensitivity of the liver and muscle to insulin, such that controlling these levels can per se reduce insulin requirement. Physiological insulin secretion is about 50–60 U/day, about half of which is basal requirement,^20^, ^21^ and this pattern is often echoed in normal‐weight people with T1DM. In T2DM however, and with gluco‐lipo‐toxicity, total insulin requirements (endogenous and injected) can be much higher—hundreds of units. Hepatic cirrhosis, endocrine conditions and therapy, and pregnancy will elevate insulin requirement, while pancreatic deficiency and renal failure reduce it.

### 2.2 Insulin therapy background

It is more important to glucose control, and thus to long‐term health outcomes, that the type(s) of insulin prescribed is properly dosed and titrated, than which of the myriad choices of marketed preparations is deployed. This is particularly true in T2DM in the overweight majority where insulin therapy is, at least in the first years after it is started, supplementary to considerable continuing pancreatic secretion. Crudely someone requiring a modest dose of basal insulin alone (say 50–60 U/day) will be still secreting 100–200 U/day, and, while islet regulation of that will be defective, it will still increase and decrease according to circulating glucose concentrations.^24^ This will partly buffer any ‘error’ in exogenous insulin dosing, day‐to‐day changes in insulin sensitivity, or variation in subcutaneous insulin absorption, provided enough injected insulin is being taken. This partly explains why hypoglycaemia is much less problem in those with the overweight insulin‐resistant phenotype, but also why it has been difficult to show improved control with modern basal insulins of more appropriate diurnal plasma profiles compared to historical intermediate‐acting insulins in treat‐to‐target clinical trials.^25^, ^26^

### 2.3 Basal insulin therapy

**FIGURE 2:**
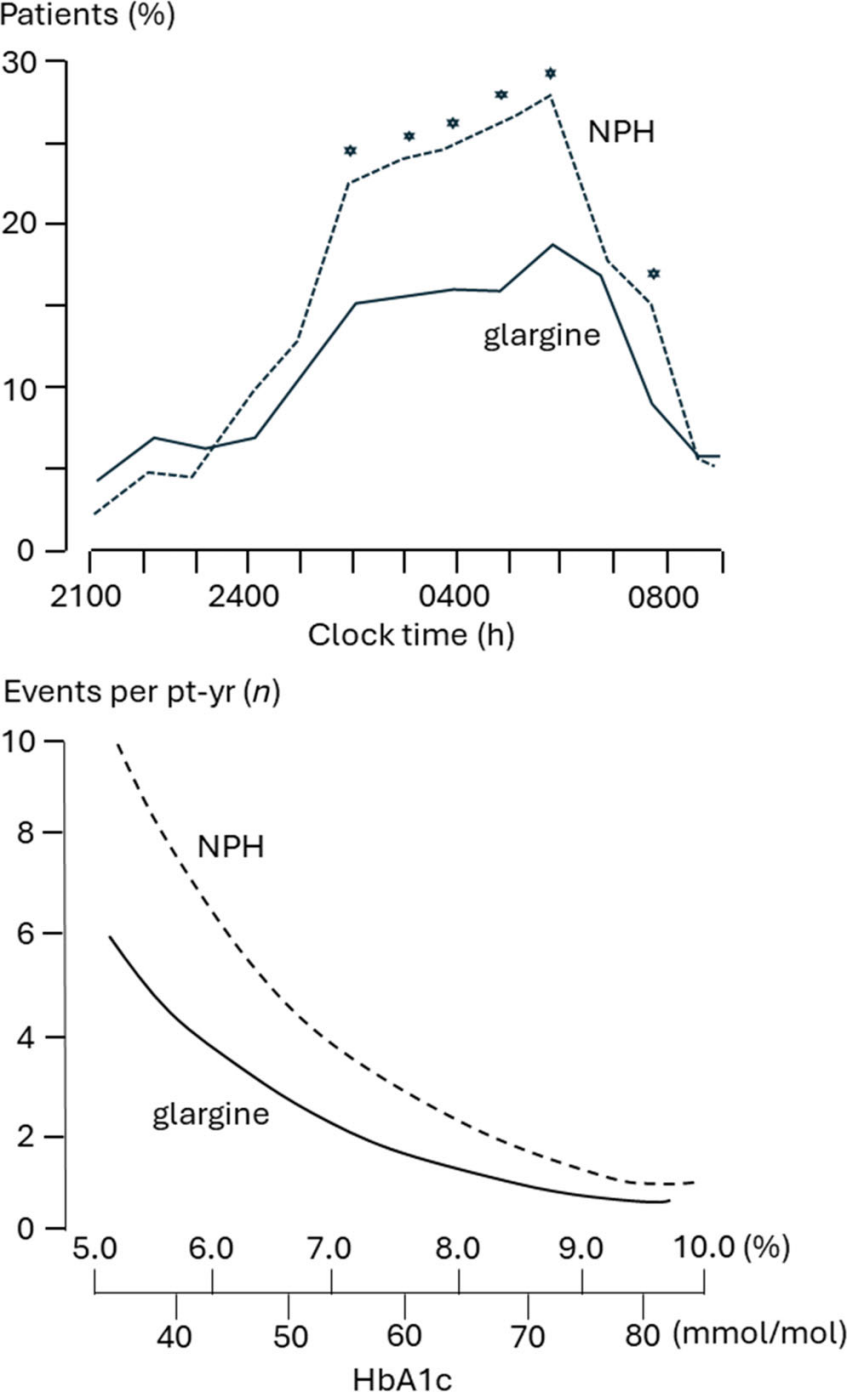
Hypoglycaemia nocturnal incidence (upper panel) and modelled study event rate (lower panel) in an RCT of once daily insulin glargine 100 U/mL versus NPH insulin in insulin naive people with type 2 diabetes.^25^ *p < 0.05. The dominant advantage of insulin glargine extends from the early to late night‐time period. It is most marked when glucose control achieves target levels. Similar findings were reported for insulin detemir.^26^

NPH insulin was described in 1946,^27^ and made available that decade. While extended‐acting, it quickly became clear from clinical experience in T1DM that its profile of action was well under 24 h, while from the 1980s the large extent of the discrepancy, with a hump of absorption at 4–6 h waning to nothing beyond 12–14 h, was described experimentally.^22^ Compared to the newer true long‐acting analogues (insulin detemir and insulin glargine), introduced this current century, this problem leads to higher risk of hypoglycaemia (Figure 2).^25^, ^26^ Accordingly, and with once‐daily administration given at bed‐time, these analogues, where affordable, came to replace NPH insulin. Indeed insulin glargine 100 U/mL (Gla‐100) has come to dominate the basal insulin market (insulin detemir is being withdrawn in some markets), and even remains a standard against which novel basal insulins (such as once weekly) are compared for regulatory purposes.^28^

Beyond Gla‐100, the basal insulin market has been complicated by the introduction of longer‐acting analogues with truly flat daily profiles (insulin glargine 300 U/mL [Gla‐300], insulin degludec [iDeg]), and the development of once‐weekly insulins.^29^ Unfortunately, the latter seems to give more hypoglycaemia, for reasons not addressed here, while the advantages of Gla‐300 and iDeg are yet again limited to reductions in the already uncommon hypoglycaemia.^29^ In T2DM a rational approach then, particularly where resources are limited (globally for the most part), is to use Gla‐100 first, and only move on if optimal control without hypoglycaemia is not achieved. However, it may be that we will find that weekly insulin injections for people using basal insulin alone will be much more acceptable as a starter insulin to people on oral agents.^30^

The second issue is combination therapy. Incretin (GLP‐1RA) injections are now generally the first injectable of choice in T2DM, having advantages over insulin in not needing feedback dose titration, being positive for weight trajectory, not themselves giving hypoglycaemia, and being widely (if expensively) available once weekly.^31^ Where they are insufficiently effective after a proper therapeutic trial, they can be used in combination with basal insulin, either as free combinations or using a fixed dose preparation. Accordingly, combinations of lixisenatide with Gla‐100, or liraglutide with iDeg, may be encountered—weekly fixed dose combinations will follow.

Basal insulin can also be combined pharmaceutically with meal‐time insulin—indeed NPH‐based premixes go back to the 1960s, and have until recently been the dominant insulin type used in south and oriental Asia. A modern version of this approach is iDeg with insulin aspart, a meal‐time analogue. The meal‐time component might be seen as advantageous if glucose levels are particularly high before starting insulin. However, the basal‐insulin‐first approach has largely displaced this class in guidelines.^32^, ^33^ Where meal‐time insulin is indicated (T1DM, pancreatic diabetes, later in the course of T2DM) independent titration of insulin dose types is required but is not possible with the combinations.

In summary, while T1DM usually requires multiple injection regimens/pumps, a useful starting point for insulin therapy in T2DM remains once daily Gla‐100, titrated (see below) against once daily pre‐breakfast self‐measured plasma glucose. The issue of insulin biosimilars is discussed in other articles in this issue, but suffice to say here that any preparation of Gla‐100 approved by a reputable regulatory authority will suffice.

### 2.4 Meal‐time insulin

The 50% of physiological insulin secretion not required for basal modulation of hepatic glucose production (HGP) is secreted for control of meal‐time glucose excursions.^20^, ^21^ It is regulated by incretins (GLP‐1, GIP) and to a lesser extent by plasma glucose.^23^ It will suppress HGP completely while promoting glucose uptake into skeletal muscle glycogen stores. As noted above people with T2DM of the overweight phenotype still excrete large amounts of their own insulin, so that if basal insulin is adequately dosed, often in combination with a GLP‐1RA incretin, prandial insulin injections will not be required until insulin secretion declines further after some years.^34^, ^35^ That however assumes the basal insulin is started appropriately, before endogenous secretion has declined too far, and before glucotoxicity and lipotoxicity further insult the islet B‐cell.

Meal‐time insulin injections give much higher excursions of circulating insulin concentrations than basal insulins. And they are just as erratically absorbed from subcutaneous tissue.^36^ So hypoglycaemia is much more common, occurring when muscle glucose uptake is over‐stimulated and HGP cannot rescue the situation through being fully suppressed despite the action of glucagon. This includes in multiple injection regimens and the use of premixes.^37^, ^38^ This issue, and the matching of insulin doses to meal size and physical activity, results in much more complex self‐management needs than for basal insulin alone, and that in turn requires a higher level of patient education and support.

Again this support and education is much more important than the exact choice of a meal‐time insulin.^31^ First‐generation meal‐time analogues do however have a significant advantage over animal and human insulins,^39^ because these latter have a prolonged tail of absorption and continue to act long after any food has been absorbed from the gut, enhancing the hypoglycaemia risk in particular into the early part of the night.^40^ Accordingly, meal‐time insulin prescriptions are now generally of an analogue, an original preparation (insulins aspart, glulisine, and lispro) or biosimilars thereof. Attempts to further improve the absorption profile of the analogues have been made, and marginal improvements are demonstrable,^41^ but in overall clinical practice the impact to date has been low, and generally their use is reserved for people with identified problems.

Meal‐time insulin formulations are solutions, enabling their use in pumps for continuous basal insulin supply. Since the same insulin is then delivering basal and meal‐time control, the choice of insulin preparation will be made on the basis of properties required for the latter; the principles given in the previous paragraph apply, typically a first‐generation insulin analogue or pump‐approved biosimilar thereof.

## 3 CLINICAL INDICATIONS FOR INSULIN THERAPY

This can be thought about in two ways, namely in what kind of clinical circumstances do specialist teams start and support insulin therapy, and in what circumstances should non‐specialists consider starting or supporting insulin therapy. In the context of this article, the first section below is then explanatory, for information for non‐specialists when seeing people with complex medical problems, while the second is intended to act as a pointer to those interested in managing routine insulin therapy.

### 3.1 T1DM, pumps, and sensors

In people with established T1DM some form of basal plus multiple meal‐time insulin therapy is the rule, to correct what is in effect absolute insulin deficiency.^42^ Occasionally, if diagnosis occurs relatively early, some kind of simpler insulin regimen (e.g., basal insulin alone) may be encountered in the early months, and rarely remission. Some forms of secondary pancreatic diabetes will need to be managed similarly, though here the absence of any glucagon secretion can predispose to more erratic control, and thus hypoglycaemia on small insulin doses. But hypoglycaemia is usual in T1DM too, reflecting the inadequacy of our current insulin delivery after >100 years of attempts to do better.^22^ The less‐than‐optimal glucose control, secondary to hypoglycaemia risk, drives the provision of insulin to the most physiologically available preparations, even where the gains with the latest analogues may be marginal.^41^ This also drives the use of insulin pumps, and the use of more sophisticated glucose‐monitoring devices including continuous glucose monitoring (CGM). The linking of CGM and pumps with sophisticated software (closed‐loop, artificial pancreas) does confer further advantage on average to glucose control profiles, and so is usually endorsed by funders.^43^

Note that quality of life healthcare gained from real‐time CGM, allowing the user to know and learn about their unpredictable glucose levels, is often larger than from pumps.^44^ CGM, like finger‐prick self‐monitoring, is calibrated to read out as plasma glucose concentration (as are laboratory measurements), although the analyte for these is interstitial SC fluid glucose and blood respectively. For CGM, this site imposes a delay in reflecting changes in circulating glucose concentration; this delay is compounded by the slow absorption of the pumped insulin analogue,^45^ and results in sub‐optimal (but better than otherwise) control in closed‐loop systems even for basal insulin delivery. Recent approaches try to compensate for this, by attempting to predict hypoglycaemia with an algorithm which detects trend, and again that has been quite successful. However, marketed closed‐loop systems are all ‘hybrid’, because no sensor‐algorithm‐pump system can begin to match the physiological incretin/secretion system. Accordingly, users still need to activate meal‐time insulin delivery, and thus estimate meal‐time insulin requirements in advance.

Familiarity with these systems is not universal even amongst specialists, and many diabetes teams will focus on one manufacturer's system or one combination of the components thereof. In a complex environment, such as someone having a medical procedure, the user will often have the expertise to advise the non‐diabetes clinical team, but where the user is unavailable (eg under general anaesthetic), or the devices unsuitable (MRI, circulatory collapse), other forms of controlled insulin administration will need to be adopted.

### 3.2 Insulin therapy in complicated clinical scenarios

A list of the common scenarios where insulin therapy may be used in people with T2DM who might otherwise not need it is given in Table 3. Such people are often under the care of another specialist team (in addition to a diabetes team) together with supportive care from primary health care. Because age is the primary risk factor for most medical conditions, such people are often in the older age groups, and often have a social care requirement in addition. Further, there usually are interactions between the therapies described for different conditions, or even complexities arising where the same therapy is prescribed for more than one condition (e.g., RAS‐blockers in cardiac/renal/diabetes medicine). Usually, here the requirement is for quite flexible insulin‐support therapy, and thus usually a multiple injection regimen is indicated (Table 3). This will also be the case in someone previously using a basal‐only insulin regimen, particularly as other glucose‐lowering medications may be regarded as contraindicated by the concomitant condition or the medications used in it. Indeed often insulin is seen as a way to simplify medical therapy in these situations.

**TABLE 3:** Medical situations in which insulin therapy is generally used and preferred over other glucose‐lowering medications in people with type 2 diabetes.

Medical condition	Comment
Inadequate glucose control in ambulatory care	After proper trial of other agents, including intolerance thereof^31^
Preference in ambulatory care	Where ‘natural’ insulin is more acceptable than ‘chemical’ medications
Acute glucose control in in‐patients (infections, myocardial infarction, major surgery, major trauma, steroid therapy, many other scenarios)	Where neglect of glucose control is found on admission or, previously adequate glucose control is compromised by a new medical event and, where this poor control may adversely affect other management Usually a multiple injection regimen, to gain control rapidly, with review of need over coming weeks back in ambulatory care
Hyperglycaemic emergencies (hyperosmolality, diabetic ketoacidosis)	Usually IV then SC insulin; doses may fall markedly over weeks as glucotoxicity wanes
New onset T2DM with marked hyperglycaemia	Rarely necessary; can give short term remission
Latent auto‐immune diabetes of adults	If ‘T2DM’ with thinner phenotype, anticipate early loss of adequate glucose control with other measures
Lean, predominantly insulin‐deficient T2DM	Often people with onset late in life; can be slow onset T1DM in disguise
Pregnancy (and pre‐pregnancy)	Tighter targets than for non‐pregnant, and should be achieved quickly; other glucose‐lowering medications often contraindicated as potentially fetotoxic (e.g., GLP‐1RA)
Short‐term control in times of metabolic stress in ambulatory care (infections, steroid therapy)	Not easy to manage, as insulin dose requirement can vary from day to day, or week to week
Co‐morbidities (kidney and liver failure, cancer, chemotherapy, other)	In renal failure often highly insulin sensitive In liver failure/cirrhosis often insulin resistant with predominant need for meal‐time insulin In cancer and many other terminal conditions insulin doses may fall away with terminal cachexia/loss of appetite; higher glucose control targets are acceptable with short‐life expectancy^31^

Abbreviations: T1DM, type 1 diabetes; T2DM, type 2 diabetes.

No single overall description of how insulin is used can suffice to cover these diverse situations. However, the underlying rule of using informed finger‐prick (self‐ or carer‐) monitoring or CGM to inform insulin dose change is fundamental. Quite rapid swings in dose requirement may occur in the face of changing metabolic stress, and medical/diabetes staff may review glucose control twice daily in some in‐patients (or even continually in intensive care). Different medical conditions, such as pregnancy, hepatic cirrhosis, and steroid therapy, may require very different ratios of mealtime to basal insulin from those experienced in standard ambulatory care (Table 3).

Some issues are common and are seen in primary care. People in hospital with metabolic stress may be started on insulin, or have their doses ramped up several fold, only for this to result in hypoglycaemia if not properly managed after discharge. Similarly, people with T2DM and a hyperglycaemic emergency will need insulin, but after discharge and with life style education may not need to continue insulin, or perhaps only basal insulin. Continued deterioration of renal function towards end‐stage disease results in increased insulin sensitivity (for still unknown reasons), and may result in dependence on insulin despite what seem like very small doses. Others will experience amelioration of insulin needs with loss of appetite as they approach end‐of‐life, whether from malignancy, heart failure or whatever. In these circumstances, glucose control targets can be relaxed,^31^ and in some with prior definite T2DM insulin may be stopped.

### 3.3 Insulin therapy in uncomplicated ambulatory care

The non‐specialist (diabetes) will encounter issues surrounding insulin therapy in three areas. The first is someone using insulin injections with problems secondary to that, and is dealt with under ‘Problems of insulin therapy’. A second is in an in‐patient or emergency department care where a professional in another speciality finds someone with diabetes taking insulin under their care; often here it will be necessary to access the expertise of the hospital's diabetes team. But also common is the situation where someone in primary or general medical care comes to the point where insulin therapy is being considered. And while the common solution is to seek specialist advice, or support from the local insulin starts team, it can also be that enough expertise is available in the current service.

**FIGURE 3:**
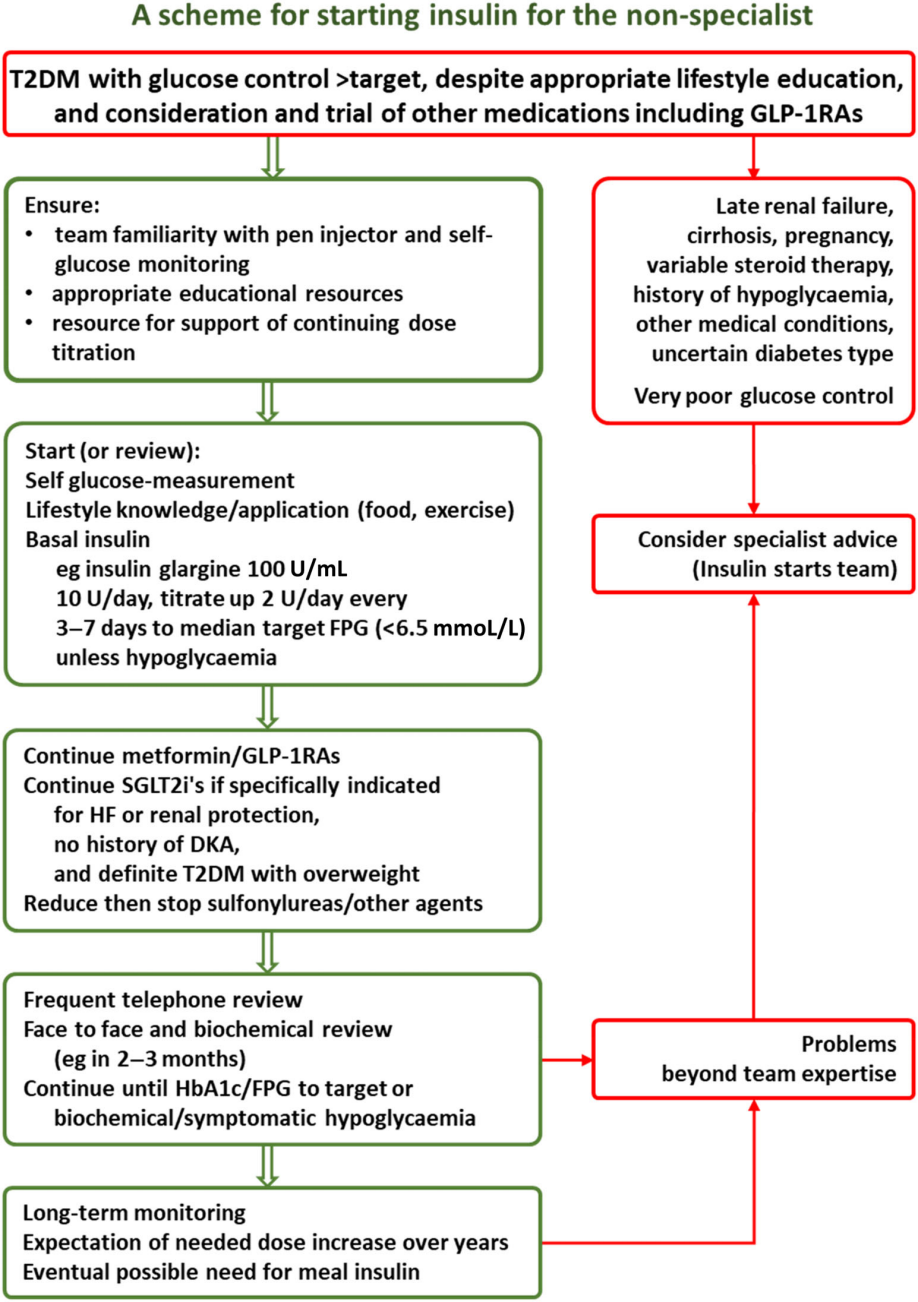
A scheme to guide starting insulin in routine ambulatory care for the non‐specialist. The dose titration regimen follows from reference 46. The scheme can be adapted to differing levels of team expertise. T2DM, type 2 diabetes.

Usually what will be considered is the use of basal insulin, as the recommended starter insulin in T2DM, often following the use or trial of a GLP‐1RA and oral agents (Figure 3).^31^, ^32^, ^33^ One relatively new advantage of this scenario is that the patient will often be familiar with injections and pen injectors, but as a minimum, the following expertise should be present in any insulin management team: (1) Familiarity with one type of insulin pen injector usually dispensing insulin Gla100—these devices are very easy to use, but there can be issues around for example storage and checking for problems before each injection, and these need to be understood and communicated. Originator and biosimilar versions are available, but the injectors are often brand‐specific, so need to be so prescribed. (2) Familiarity with a finger‐prick self‐monitoring system—devices from the major long‐established manufacturers are recommended as more reliably accurate and precise. Again such familiarity is easily gained, but must be enough to instil confidence in prospective users. (3) Materials to support a simple continuing dose‐escalation algorithm such as a starting dose of 10 U/day and escalation every 2–3 days by 2 U if no evidence of hypoglycaemic glucose levels,^46^ and the expectation that support for this will last a small number of months. (4) Other diabetes education facilities and materials; will be present from earlier activities of the diabetes team, but issues around calorie intake and risk of hypoglycaemia will need to be addressed again for emphasis, as well as foreign travel and sick day rules.

It is not enough simply to start inulin. The final dose expectation will be around 30–80 U/day taken late evening, with a range of 10–150 U/day, so may only be reached after some months. Given human nature continued supportive contact is needed to ensure continued dose titration (Figure 3). Once the FPG target is achieved, active titration will cease, but with the expectation of a slow increment in dose over the years as endogenous insulin secretion further declines. Concomitant glucose‐lowering medication should be tailed off, with the exception of metformin and GLP‐1RAs if tolerated—beware of precipitating a rapid deterioration in glucose control before an adequate insulin dose is achieved (Figure 3).

Moving on in months or years when pre‐breakfast glucose control cannot be achieved despite the pushing of basal insulin doses to high levels (>150 U/day), or where problems such as hypoglycaemia intervene, does require more expertise than in usual outside diabetes specialist teams. This then would include the use of meal‐time insulins.

## 4 MANAGING PROBLEMS OF INSULIN THERAPY

In terms of off‐target side effects, insulin, a natural hormone, is very well tolerated—the injection/infusion site needs to be rotated within one particular area (such as the anterior abdominal wall) to avoid scarring and local metabolic effects (lipohypertrophy). Allergic problems are vanishingly rare with modern insulins. The barrier to starting an injectable is real, often compounded by insulin stigma as a result of its association with more advanced ‘disease’.^47^ If started without proper continuing support, failure of persistence of use, and poor adherence to daily injections, are not unusual.^48^ Two issues (below) are however connected to the mode of action of insulin.

### 4.1 Body weight gain

As hyperglycaemia is improved, glycosuria is ameliorated, calorie balance becomes relatively positive, and those calories will have to go to storage as fat, unless calorie intake is reduced.^49^, ^50^ Furthermore high circulating glucose concentrations drive the rate of its own metabolism by a mass action effect, so again amelioration of that reduces calorie disposal, and again the excess can only go to fat.^49^, ^50^ Other substrates, such as fatty acids, are similarly affected.^49^ These effects will cause most weight gain in those whose glucose control is initially poorest, and in those whose control is then most improved. Since insulin is often started late and since it is so effective, the risk of weight gain is higher than with other glucose‐lowering medications. In ORIGIN (people started on insulin before other agents) weight gain was 1.6 kg in 5 years, and in the UKPDS (starting soon after diagnosis) 4.0 kg in 10 years, neither greatly different from age trends.^6^, ^51^

The problem can thus be ameliorated by starting insulin before control is too poor, and by prospective education on the reasons for weight gain, and setting expectations in the context of the review of calorie intake.

### 4.2 Managing hypoglycaemia

Hypoglycaemia is important—indeed without hypoglycaemia insulin doses could be titrated to normoglycaemia, and the risk of vascular complications from high glucose levels would vanish. It is also important because it can be unpleasant and inconvenient, and, if cerebral dysfunction occurs, dangerous. Plasma glucose levels of <3.0 mmol/L are now regarded as a ‘clinical risk’ but any below normal are probably predictive of severe hypoglycaemia, defined as requiring help from others.^52^, ^53^ But it is a much bigger problem in T1DM (nearly universal in any year) than in T2DM. In the T2DM GRADE study insulin glargine resulted in an excess over liraglutide/sitagliptin of 12.4% of participants for any low glucose level over 5 years, and 0.5% excess for severe hypoglycaemia—figures which were lower than for the sulfonylurea glimepiride.^54^, ^55^ If, as is the case in T2DM, insulin is only supplementary to endogenous production, then the opportunity for excess from erratic SC absorption is mostly mitigated, and glucose production (liver) will not be oversuppressed nor glucose uptake (muscle) overstimulated. Further overweight people with T2DM are universally insulin resistant—and that also mitigates the effect of insulin excess. Unlike the situation in some people with T1DM or pancreatic DM, glucagon secretion is preserved though dysfunctional, so providing the system with some means of countering hypoglycaemia.

**FIGURE 4:**
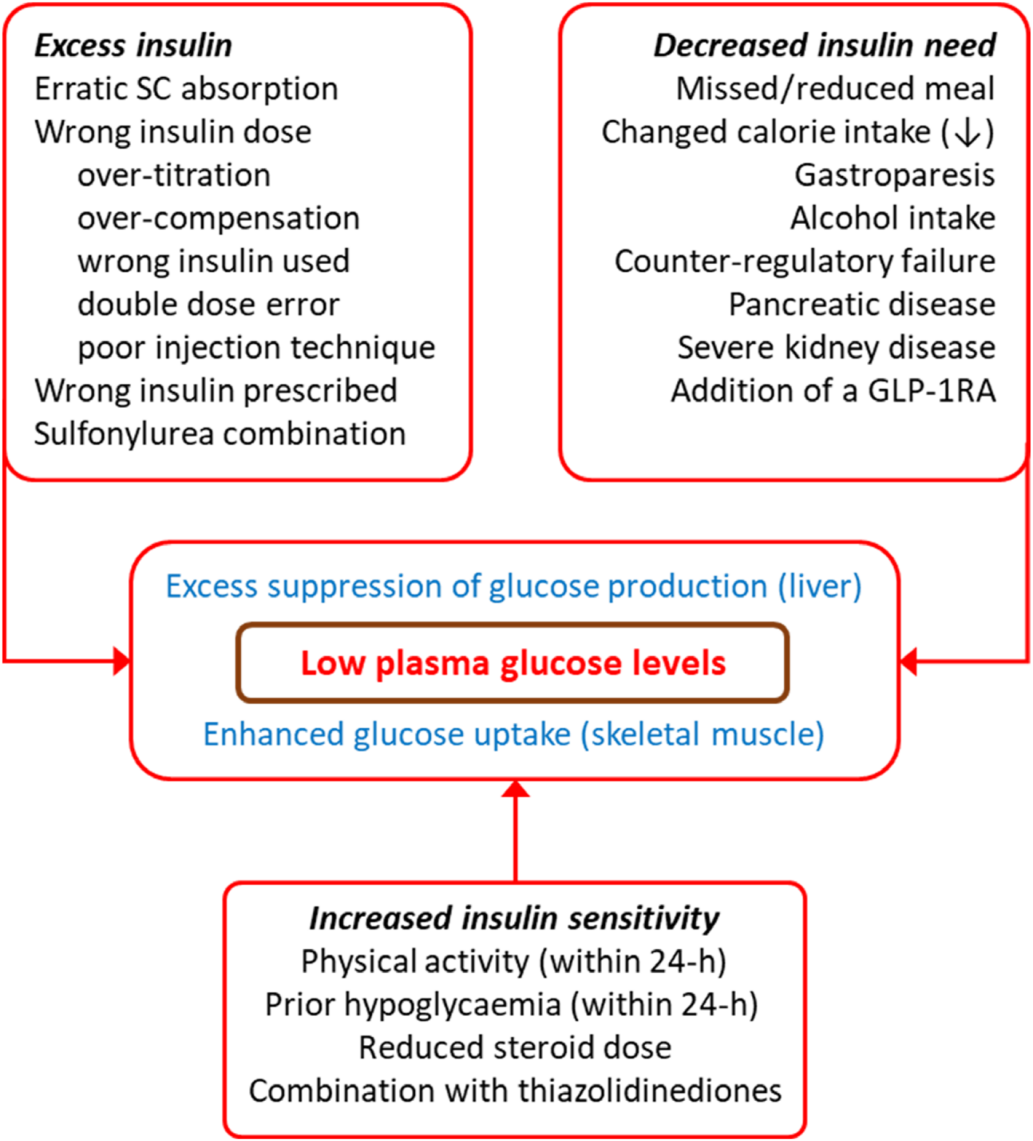
Schema to illustrate how excess insulin supply, decreased insulin need, and increased insulin sensitivity can contribute, singly or in combination, to the genesis of an episode of hypoglycaemia. Some rarer contributions to the three domains are omitted.

Nevertheless, because hypoglycaemia is spasmodic, and more of a problem where insulin doses are unstable, it does present to the non‐specialist. An approach to guide clinical management will include the domains in Table 4. Enquiry will need to be made to detect possible changes in insulin sensitivity (unusual physical activity in the previous 24 h), changes in calorie consumption including missed meals (especially for those using meal‐time insulin), alcohol consumption, and the possibility of erroneous dose administration either mistakenly or through inappropriate dose adjustment, this sometimes as an in‐patient (Figure 4). Occasionally insulin types may be mixed up, or even confused with those of another patient. Such enquiry will be followed by review of self‐monitoring records, which may reveal inappropriate insulin dosage, or erratic control. Identification of an issue may suggest advice alone is needed, but insulin dose reductions may be required if monitoring suggests a continuing problem. These can be bespoke—tailored around episodes of exercise or periodic changes in eating habits for example.

**TABLE 4:** Hypoglycaemia: Outline scheme for the non‐specialist in understanding and managing commoner origins of hypoglycaemia.

Background information	Domains of information	Comment
Type/aetiology of diabetes	T1DM	Usual in any month/year
	Pancreatic (secondary)	Erratic glucose levels usual
	T2DM	Minority of people affected
Lifestyle (general)	Erratic or consistent	Often correlates with younger age
		May become erratic with frailty
Insulins used	Basal ± meal‐time	Meal‐time insulins much greater problem
	Human/animal	Greater problem than with analogues
	Second generation analogues	Marginally superior to first
	Weekly insulins	Somewhat inferior
	Insulin regimen	Unbalanced over day; meal versus basal ratio
Glucose measurements	SMPG or CGM problems	Review performance in real time
Non‐specific symptoms	Other medical conditions	Confirmation with self‐measurements?
Medical conditions	Renal failure—late or end stage	Erratic control at low insulin doses
	Change in steroid therapy	
	Recovery from metabolic stress	Trauma, surgery, ketoacidosis
	Pregnancy, post‐pregnancy	

Acute issues	Circumstance	Comment
Physical activity	At the time of hypo	Lowers glucose unless high already
	In previous 24 h	Causes higher insulin sensitivity
	Change in circumstance	Holidays; hospitalization and post‐hospitalization
Food and drink	Missed or reduced meal	
	Reduction in high‐CHO drinks	Such as colas, beer
	Change (increase) in alcohol intake	Notably spirits
Insulin	Confused/mistaken insulin doses/types	Review insulin devices and use
	Inappropriate insulin dosage	Review self‐measured results

*Note*: This table is not intended to be comprehensive and omits for example many medication interactions, factitious and behavioural issues, and hypoglycaemia of other origin.

Abbreviations: CGM, continuous glucose monitoring; CHO, carbohydrate; SMPG, self‐measured plasma glucose; T1DM, type 1 diabetes; T2DM, type 2 diabetes.

As is usual with insulin therapy, a problem beyond personal expertise will suggest need for advice from an insulin management team.

## 5 CONCLUSIONS

It is clear that the non‐specialist must have a role in the management of insulin therapy, because all will see people using insulin. While it is acknowledged that insulin therapy can be tricky, thanks to differences in insulin sensitivity between individuals and thus the need for dose titration, and because both human lifestyles and SC insulin absorption are erratic, the simple concept of basal and meal‐time insulin secretion, matched to the provision of basal and meal‐time insulin preparations, means that the knowledge barriers to advising on insulin therapy need not be high.

This is particularly true for basal insulin in T2DM as a starter insulin, whether as first injectable or with/after a GLP‐1RA. Here the clinical advisor needs only familiarity with a pen injector and self‐measurement device, and similarly with a simple dose titration algorithm. This is not a say that this is a ‘provide and forget’ prescription—continued support lessening after a few months is critical. Support from an insulin starts team can be very helpful.

Every health practitioner has limitations to their expertise. In more complicated scenarios as with much in‐patient care, or where complicated by other medications, it is likely that specialist care will need to be requested. Specialists should ensure that is accessible.

## FUNDING INFORMATION

The funding for this special issue was not known to the author throughout the writing and publication process. No other funding was utilized.

## CONFLICT OF INTEREST STATEMENT

The author receives research support from Sanofi, and has consulted personally or through his institution for Novo Nordisk, Eli Lilly, Mylan/Viatris/Biocon, and Gan and Lee, as well as the manufacturers of competing non‐insulin glucose‐lowering medications.

## PEER REVIEW

The peer review history for this article is available at https://www.webofscience.com/api/gateway/wos/peer-review/10.1111/dom.16280.

## Data Availability

No new author's data included in this review.
